# Medullary Noradrenergic Neurons Mediate Hemodynamic Responses to Osmotic and Volume Challenges

**DOI:** 10.3389/fphys.2021.649535

**Published:** 2021-04-23

**Authors:** Stefanne Madalena Marques, Lara Marques Naves, Talita de Melo e Silva, Keilah Valéria Naves Cavalcante, Juliana Milan Alves, Marcos Luiz Ferreira-Neto, Carlos Henrique de Castro, Andre Henrique Freiria-Oliveira, James Oluwagbamigbe Fajemiroye, Rodrigo Mello Gomes, Eduardo Colombari, Carlos Henrique Xavier, Gustavo Rodrigues Pedrino

**Affiliations:** ^1^Department of Physiology, Biological Sciences Institute, Federal University of Goiás, Goiânia, Brazil; ^2^Department of Physiology and Biophysics, Institute of Biomedical Science, University of São Paulo, São Paulo, Brazil; ^3^Department of Physiology, Institute of Biomedical Sciences, Federal University of Uberlândia, Uberlândia, Brazil; ^4^Department of Physiology and Pathology, School of Dentistry, São Paulo State University (UNESP), Araraquara, Brazil

**Keywords:** central nervous system, resuscitation, lesion, blood pressure, hypertonic saline

## Abstract

Despite being involved in homeostatic control and hydro-electrolyte balance, the contribution of medullary (A1 and A2) noradrenergic neurons to the hypertonic saline infusion (HSI)-induced cardiovascular response after hypotensive hemorrhage (HH) remains to be clarified. Hence, the present study sought to determine the role of noradrenergic neurons in HSI-induced hemodynamic recovery in male Wistar rats (290–320 g) with HH. Medullary catecholaminergic neurons were lesioned by nanoinjection of antidopamine-β-hydroxylase–saporin (0.105 ng·nl^−1^) into A1, A2, or both (LES A1; LES A2; or LES A1+A2, respectively). Sham rats received nanoinjections of free saporin in the same regions (SHAM A1; SHAM A2; or SHAM A1+A2, respectively). After 15 days, rats were anesthetized and instrumented for cardiovascular recordings. Following 10 min of stabilization, HH was performed by withdrawing arterial blood until mean arterial pressure (MAP) reaches 60 mmHg. Subsequently, HSI was performed (NaCl 3 M; 1.8 ml·kg^−1^, i.v.). The HH procedure caused hypotension and bradycardia and reduced renal, aortic, and hind limb blood flows (RBF, ABF, and HBF). The HSI restored MAP, heart rate (HR), and RBF to baseline values in the SHAM, LES A1, and LES A2 groups. However, concomitant A1 and A2 lesions impaired this recovery, as demonstrated by the abolishment of MAP, RBF, and ABF responses. Although lesioning of only a group of neurons (A1 or A2) was unable to prevent HSI-induced recovery of cardiovascular parameters after hemorrhage, lesions of both A1 and A2 made this response unfeasible. These findings show that together the A1 and A2 neurons are essential to HSI-induced cardiovascular recovery in hypovolemia. By implication, simultaneous A1 and A2 dysfunctions could impair the efficacy of HSI-induced recovery during hemorrhage.

## Introduction

Hemorrhagic trauma is a leading cause of mortality and morbidity in people below 44 years old (Curry and Davis, 2012). Studies have shown that hemorrhagic shock is responsible for about 40% of trauma-related deaths (Kauvar et al., 2006). Hemorrhagic shock often promotes hypoperfusion, reduction in oxygen distribution, decrease in preload, and cardiac output (Xu et al., 2013). Although the hemorrhage control and blood volume replacement are an important therapeutic intervention, a better understanding on the pathophysiological mechanisms composing hypovolemic shock could engender efficient treatment.

Hyperosmolarity induced by intravenous hypertonic saline infusion (HSI) solution (0.3 M of NaCl) is an efficient strategy to suppress hemorrhagic shock (Krausz, 2006). Studies have shown improvements in cardiovascular and immunological parameters after HSI during hypovolemic state, sepsis, or traumatic injury (Velasco et al., 1980; Lopes et al., 1981; Kramer, 2003). The cardiovascular effects triggered from HSI go far beyond plasma volume expansion (Walsh and Kramer, 1991). The HSI-induced improvement in cardiovascular function during hemorrhage partially depends on the central nervous system (CNS) activation (de Almeida Costa et al., 2009). Recently, it was demonstrated that the blood pressure restoration after HSI-induced hypotensive hemorrhage (HH) was blocked by denervation of the carotid body chemoreceptors (Pedrino et al., 2011).

Neuroanatomical, neurophysiological, and pharmacological studies have provided robust evidence on the role of nucleus of the solitary tract (NTS), caudal ventrolateral medulla (CVLM) region, and rostral ventrolateral medulla (RVLM) neurons in cardiovascular and respiratory regulation (Aicher et al., 1995, 1996, 2000; Blanch et al., 2007). The A1 and A2 noradrenergic neurons located at CVLM and NTS, respectively, project to hypothalamic regions such as the subfornical organ (SFO), the paraventricular nucleus (PVN), the supraoptic nucleus (SON), and the median preoptic nucleus (MnPO) to promote cardiovascular and blood volume regulation (Tanaka et al., 1997; Guyenet, 2006; Menani et al., 2014b).

Therefore, the hypothesis herein raised is that the integrity of A1 and A2 neurons would be required to reach the hemodynamic improvements induced by HSI-induced during HH. Hence, by lesioning A1 or A2 noradrenergic neuronal groups, the present study sought to evaluate the participation of these nuclei in the HSI-induced cardiovascular recovery after hemorrhage.

## Methods

### Animals

All experiments were conducted on adult male Wistar rats aged 8–10 weeks (290–320 g) obtained from the central animal facility of the Federal University of Goias. These animals were housed at the Department of Physiological Sciences, in polypropylene cages (45 cm × 30 cm × 15 cm) under controlled conditions of 12-h light–dark cycle (07:00 a.m. to 07:00 p.m.) and temperature (22.0 ± 2°C). All experimental procedures follow the rules and Guidelines for Care and Use of Laboratory Animals as approved by the Ethics Committee of the Federal University of Goias (protocol number 034/12).

### Experimental Groups

The rats were split into six experimental groups. In one set of surgeries, the catecholaminergic neuron lesion was induced by nanoinjecting the conjugated anti-DβH-saporin to achieve the following: (i) A1 lesion (LES A1; *n* = 8); (ii) A2 lesion (LES A2; *n* = 8); and (iii) A1 and A2 concomitant lesion (LES A1+A2; *n* = 6). In another set of surgeries, animals were subjected to nanoinjection of (non-conjugated) saporin to achieve the following: (i) A1 (SHAM A1; *n* = 8); (ii) A2 SHAM A2; *n* = 6); and (iii) A1 and A2 neurons (SHAM A1+A2; *n* = 7).

### Nanoinjections of Anti-DβH-Saporin or Saporin Into the Caudal Ventrolateral Medulla and/or Nucleus of the Solitary Tract

Animals were anesthetized (ketamine 10%, 1 ml·kg^−1^; and xylazine 2%, 0.7 ml·kg^−1^; Syntec, Santana de Parnaíba, SP, Brazil) prior to their ventral positioning in the stereotaxic apparatus (David Kopf Instruments, Tujunga, CA, USA). The incisor bar was set at 11 mm below the interaural line. After partial removal of the occipital bone, the meninges covering the dorsal surface of the brainstem were carefully cut to visualize the *calamus scriptorius* (the anatomical setpoint for coordinates). The anti-DβH-saporin (100 nl, 0.105 ng·nl^−1^; Advanced Targeting Systems, San Diego, CA, USA) or an equimolar dose of saporin (100 nl, 0.022 ng·nl^−1^; Advanced Targeting Systems, San Diego, CA, USA) was injected bilaterally into the CVLM and/or NTS. Glass micropipette was positioned at 0.3 mm rostral and 0.3 mm caudal from the calamus scriptorius, 1.8 mm lateral from the midline, and 1.8 mm ventral from the dorsal surface to achieve nanoinjections into the CVLM. The NTS nanoinjections involve placement of a glass micropipette at 0.0 and −0.5 mm caudal to calamus scriptorius, 0.0 mm lateral from the midline, and 0.5 mm ventral from the dorsal surface. These coordinates are based on the region of the CVLM (A1 neurons) and NTS (A2 neurons) (Pedrino et al., 2012; da Silva et al., 2013). After each nanoliter injection, the micropipette was left in place for 3 min to ensure that the volume was delivered into the desired site. Subsequently, the incision was sutured, and the animals were placed on a heated pad to maintain their body temperature during surgical recovery. A prophylactic antibiotic dose (penicillin, 60,000 IU·kg^−1^, i.m.; Sigma-Aldrich, St. Louis, MO, USA) was injected after surgery. All experimental procedures were performed 15 days after the nanoinjections to ensure animal recovery and establishment of the neuronal lesion (Pedrino et al., 2012).

### Surgical Procedures

The animals were subjected to anesthetic induction through the administration of halothane (2%, Tanohalo, Cristália, Itapira, SP, Brazil) in 100% O_2_ prior to the catheterization of femoral artery and vein. After vein catheterization, anesthesia was maintained by administration of urethane (1.2 g·kg^−1^, i.v.; Sigma-Aldrich, St. Louis, MO, USA). Additional catheter was inserted into the right carotid artery to withdraw blood during HH. Tracheostomy was performed to reduce airway resistance. The renal blood flow (RBF) and aortic blood flow (ABF) baseline were recorded through a miniature probe that was placed around the left renal artery and abdominal aorta. The hind limb blood flow (HBF) was calculated by using the following equation: (ABF) – (2 × RBF). The body temperature of rats was maintained between 36 and 37°C on a heating pad throughout the experimental procedures.

### Recording of Cardiovascular Parameters

The pulsatile arterial pressure (PAP) of the anesthetized animal was recorded continuously through the arterial cannula that was connected to an amplifier-coupled pressure transducer (Bridge Amp FE221; ADInstruments, Colorado Springs, CO, USA). Data were digitized at a frequency of 2,000 samples·s^−1^ using a digital analog converter (PowerLab 4/25, ML845, ADInstruments, Colorado Springs, CO, USA). The MAP was calculated from the integral of PAP signal using the LabChart software (v.7.3.7, ADInstruments, Colorado Springs, CO, USA), while heart rate (HR) was calculated as instantaneous frequency from the PAP signal (v.7.3.7, ADInstruments, Colorado Springs, CO, USA). The RBF and ABF were recorded through a miniature probe placed around the left renal artery and the abdominal aorta just before the renal artery. Miniature probes were connected to a T206 flowmeter (Transonic Systems, Inc., Ithaca, NY, USA), which determines the flow rate in absolute values (ml·min^−1^). The signals obtained were transferred to the acquisition and data analysis MP150 system (PowerLab 4/25, ML845, ADInstruments, Colorado Springs, CO, USA). Data were digitized at a sampling frequency of 1,000 samples·s^−1^. The HBF was calculated by using the following equation: (ABF) – (2 × RBF). Changes in RBF, ABF, and HBF were calculated as the absolute change to baseline (ΔRBF, ΔABF, and ΔHBF, respectively).

The total renal, aortic, and posterior limb blood volume after the HSI was calculated as the summation of the mean blood flow values obtained in every minute during recovery period (60 min) using the LabChart software (v.7.3.7, ADInstruments, Colorado Springs, CO, USA) analysis.

### Hypotensive Hemorrhage and Sodium Overload

After 10 min of stabilizing the baseline of cardiovascular parameters, blood was withdrawn slowly for 10 min until the MAP levels reached 60 mmHg to establish HH. This arterial blood pressure was maintained for 10 min prior to HSI (3 M of NaCl) into the catheterized right femoral vein (60 s; 1.8 ml·kg^−1^ b.wt.).

### Histology and Immunohistochemistry

At the end of the experiments, transcardiac perfusion with heparinized (5,000 UI L^−1^) saline (0.15 M of NaCl; 200 ml) and a solution of paraformaldehyde (0.2 M; 500 ml; Sigma-Aldrich, St. Louis, MO, USA) in sodium phosphate buffer (pH 7.4) was carried out. The brain of each rat was removed and fixed in paraformaldehyde (0.2 M; 30 ml) solution for 1–2 h and then cryoprotected in sucrose solution (0.8 M; 30 ml; Sigma-Aldrich, St. Louis, MO, USA). Coronal sections (40 μm thick) of brainstem were performed with freezing microtome (CM1860, Leica, Deutsch, Germany). The sections were collected (in four serially adjacent sets) and stored in sodium phosphate-buffered saline (PBS) (0.02 M, Sigma-Aldrich, St. Louis, MO, USA; pH 7.4).

Immunohistochemical detection of tyrosine hydroxylase (TH; catecholamine synthesis enzyme) was carried out on each of the fourth brainstem section. The sections were washed in ImmunoBuffer (IB; Triton 0.3% in PBS; Sigma-Aldrich, St. Louis, MO, USA) prior to a 30 min incubation in 2% normal horse serum (Vector Laboratories Inc., Burlingame, CA, USA) in IB. The sections were incubated overnight with TH mouse monoclonal antibody (1:2,000 dilution, Catalog # 22941; Lot # 1602001, clonality mouse monoclonal, sotype IgG1, Immuno Star Inc., Hudson, WI, USA) with 2% normal horse serum in IB prior to an overnight incubation with biotinylated horse anti-mouse IgG (1:500 dilution, Vector Laboratories Inc., Burlingame, CA, USA). After these incubations, the sections were processed with the avidin–biotin procedure by using Elite Vectastain reagents (Vector Laboratories Inc., Burlingame, CA, USA). Diaminobenzidine (DAB) was used to produce a brown product of cytoplasmic TH reaction. The sections were set on slides, dehydrated in alcohols, and clarified in xylene with the glass slide or coverslip.

### Count of Neurons

The TH-labeled neurons were counted in each of the fourth section of medulla (40 of each 160 μm). All cells with cell body and at least one dendrite or axon in the VLM (A1/C1 neuronal clusters) and NTS (A2/C2 neuronal clusters) were counted bilaterally to quantify the extent of anti-DβH-saporin-induced lesion. The neurons (at the magnification of 200×) were counted with the aid of an optical microscope (Leica DM500, Leica Microsystems, SP, Brazil) coupled to a digital image acquisition system (Leica Application Suite, V.3.10, Leica Microsystems, Brazil).

### Statistical Analysis

The GraphPad Prism software (v.6; GraphPad Software, Inc., La Jolla, CA, USA) was used for the statistical analysis of experimental data. Cardiovascular parameters were expressed as mean ± standard error of the mean (SEM). The MAP, HR, RBF, ABF, and HBF variations were analyzed using two-way analysis of variance (ANOVA) followed by Bonferroni *post-hoc* test. The effects of anti-DβH-saporin or saporin treatment on the number of catecholaminergic medullary neurons were presented as means ± SEM. The number of cells in each section was compared by a one-way ANOVA and Bonferroni *post-hoc* test. Additionally, the total cell counts for the A1, C1, A2, and C2 regions were calculated and compared by using unpaired Student's *t*-tests. Differences were considered significant at *p* < 0.05.

## Results

### Effects of A1 Neuronal Lesions on Hypertonic Saline Infusion-Induced Cardiovascular Responses in Rats With Hypotensive Hemorrhage

**Figure 1:**
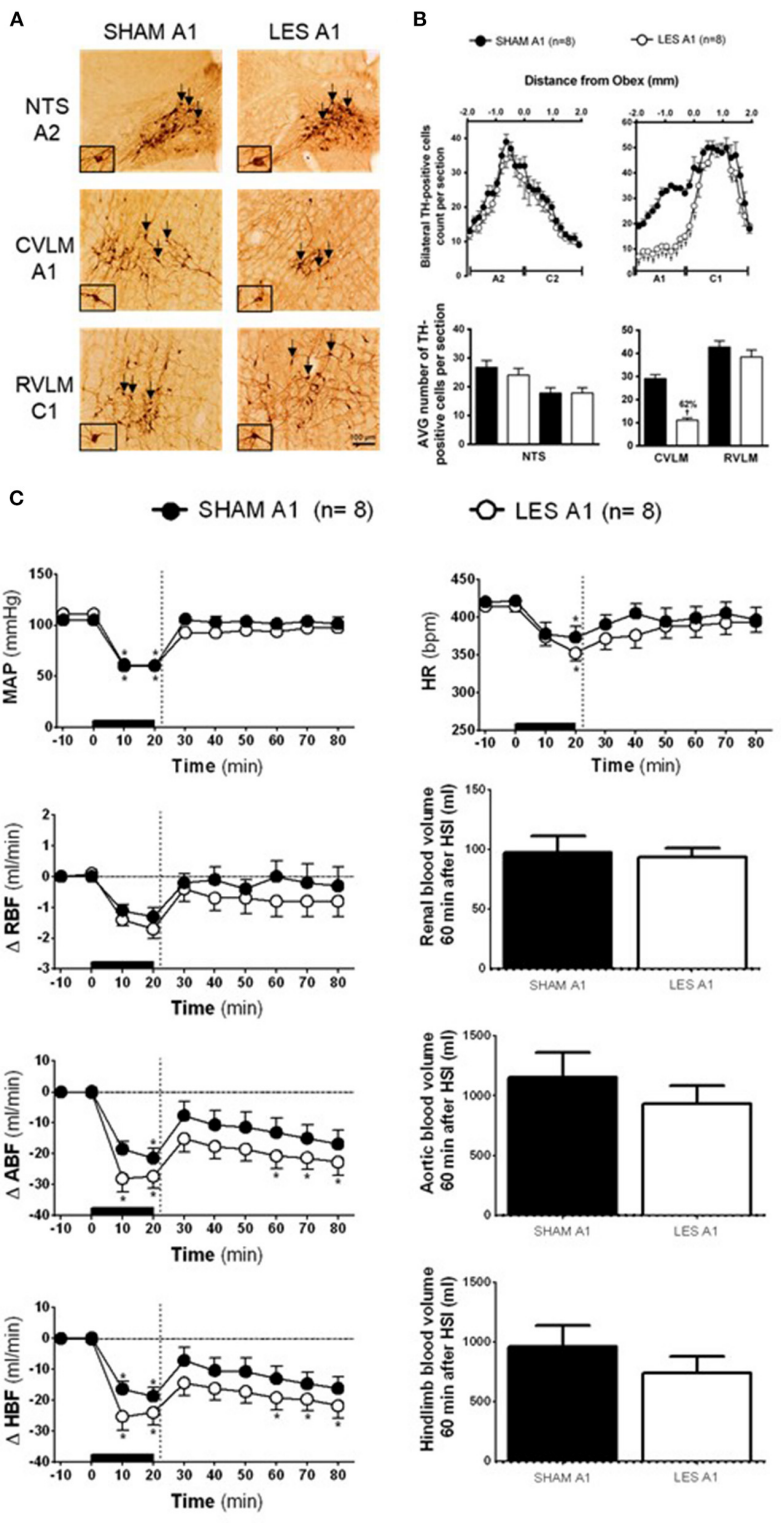
**(A**) A representative photomicrograph of medullary coronal sections (40 μm) with the scale bar of 100 μm. The arrows indicate positive tyrosine hydroxylase neurons in the nucleus of the solitary tract (NTS—cluster A2), caudal ventrolateral medulla (CVLM—cluster A1), and rostral ventrolateral medulla region (RVLM—cluster C1) of the animals bilaterally nanoinjected with saporin (SHAM A1 group) or saporin-anti-DβH (LES A1) in CVLM. **(B)** Quantification of tyrosine hydroxylase (TH)-positive cells in the medullary regions. The number of positive TH cells in the nucleus of the solitary tract (NTS—clusters A2 and C2), caudal ventrolateral medulla (CVLM—cluster A1), and rostral ventrolateral medulla region (RVLM—cluster C1) of the animals bilaterally nanoinjected with saporin (SHAM A1 group) or saporin-anti-DβH (LES A1) into CVLM. **(C)** The effects of A1 catecholaminergic neuron lesions on changes in mean arterial pressure (MAP), heart rate (HR), variations of renal blood flow (ΔRBF, ml·min^−1^), aortic blood flow (ΔABF, ml·min^−1^), hind limb blood flow (ΔHBF, ml·min^−1^), renal blood volume (RBV), aortic blood volume (ABV), and hind limb blood volume (HBV) during 60 min after hypertonic saline infusion (HSI) in the SHAM A1 (*n* = 8) and LES A1 (*n* = 8) groups submitted to hypotensive hemorrhage. Data expressed as means ± standard error of the mean (SEM) by two-way ANOVA and Bonferroni *post-hoc* test. ^†^*p* < 0.05 vs. control group; *p < 0.05 vs. time 0. Black bar and dashed line represent hemorrhagic period and HSI (3 M of NaCl), respectively.

TH-positive neurons were found within the VLM and NTS at ~1,900 μm caudal and 1,900 μm rostral from the obex (Figures 1A,B). In the CVLM region (SHAM A1; *n* = 8), the average number of TH-positive neurons was ~29 cells per section. The nanoinjection of anti-DβH-saporin conjugated into CVLM region (A1 lesioned group; *n* = 8) reduced the average number of TH-positive neurons caudal to the obex to ~11 cells per section. The number of TH-positive cells was reduced by 62% in the region of A1 neuronal cluster (Figures 1A,B). The nanoinjections of anti-DβH-saporin in the CVLM region did not reduce the average number of TH-positive cells in the region of RVLM (C1) neurons (Figures 1A,B). The number of TH-positive NTS (A2) and NTS (C2) neurons was not changed significantly (Figures 1A,B).

The SHAM A1 and A1 lesioned (LES A1) groups showed similar baseline values of MAP, HR, RBF, ABF, and HBF (Table 1). The volume of blood that was drawn to keep MAP at 60 mmHg during the hemorrhage was also similar in both groups (SHAM A1: 3.8 ± 0.2 ml vs. LES A1: 3.5 ± 0.6 ml).

**Table 1:** Baseline values of MAP, HR, RBF, ABF, and HBF.

**Group**	***N***	**MAP** **(mmHg)**	**HR** **(bpm)**	**RBF** **(ml·min^**−1**^)**	**ABF** **(ml·min^**−1**^)**	**HBF** **(ml·min^**−1**^)**
SHAM A1	8	105.3 ± 2.8	413.8 ± 17.9	2.4 ± 0.2	39.4 ± 2.9	34.1 ± 2.6
LES A1	8	111.0 ± 3.9	415.0 ± 7.6	3.0 ± 0.4	41.4 ± 6.9	35.6 ± 6.1
SHAM A2	6	108.7 ± 5.4	394.6 ± 12.4	3.5 ± 0.8	46.3 ± 8.1	39.3 ± 7.5
LES A2	8	108.3 ± 1.9	403.8 ± 12.7	3.1 ± 0.4	45.3 ± 3.6	39.2 ± 3.6
SHAM A1+A2	7	114.7 ± 5.8	396.8 ± 19.5	3.0 ± 0.4	45.2 ± 6.9	39.1 ± 7.5
LES A1+A2	6	109.1 ± 7.9	413.6 ± 8.6	3.7 ± 0.3	47.8 ± 8.2	40.4 ± 9.0

*Values are means ± standard error of mean (SEM)*.

*MAP, mean arterial pressure; HR, heart rate; RBF, renal blood flow; ABF, aortic blood flow; HBF, hind limb blood flow*.

The magnitude of the hypotension provoked by hemorrhage was similar in the SHAM A1 (105.3 ± 2.8 to 60.5 ± 0.5 mmHg) and LES A1 groups (111.1 ± 3.9 to 60.4 ± 0.3 mmHg; following 20 min of hemorrhage; Figure 1C). Figure 1C shows restoration of MAP to baseline values in both SHAM A1 (103.9 ± 4.4 mmHg) and LES A1 (95.2 ± 3.9 mmHg) after 30 min of HSI. The pressor restoration remained stable during 60 min after HSI (SHAM A1: 101.4 ± 6.6 mmHg vs. LES A1: 97.8 ± 2.6 mmHg; Figure 1C). The HR responses did not differ between the SHAM A1 and LES A1 groups during the HH. The bradycardia was evident after 20 min of hemorrhage (SHAM A1: 373.6 ± 24.4 bpm and LES A1: 352.6 ± 10.6 bpm; *p* < 0.05; Figure 1C). After 10 min of HSI, the SHAM A1 and LES A1 groups recovered from hemorrhage-induced changes in HR (390.7 ± 13.4 and 371.8 ± 15.1 bpm, respectively; Figure 1C). This response was maintained during the 60 min (396.7 ± 16.6 and 394.3 ± 12.2 bpm, respectively; Figure 1C).

During hemorrhage, RBF was reduced in both groups (SHAM A1: −1.3 ± 0.3 ml·min^−1^ vs. LES A1: −1.7 ± 0.3 ml·min^−1^; after 20 min of hemorrhage; *p* < 0.05; Figure 1C). After the HSI, the RBF returned to baseline (SHAM A1: −0.4 ± 0.3 ml·min^−1^ vs. LES A1: −0.7 ± 0.5 ml·min^−1^; 60 min after HSI; Figure 1C).

Hemorrhage induced equipotent decrease in the ABF in the SHAM A1 (−21.4 ± 3.1 ml·min^−1^) and LES A1 groups (−27.3 ± 3.9 ml·min^−1^; after 20 min of hemorrhage; *p* < 0.05; Figure 1C). Subsequent restoration of this parameter to basal level was demonstrated in the SHAM A1 group (−16.8 ± 4.4 ml·min^−1^; after 60 min of HSI; Figure 1C). In the LES A1 group, HSI did not restore baseline values of ABF (−22.7 ± 4.2 ml·min^−1^; after 60 min of HSI; *p* < 0.05; Figure 1C). No significant differences could be seen between two groups. We observed a similar decrease in the HBF during HH in the SHAM A1 (−18.7 ± 2.8 ml·min^−1^) and LES A1 groups (−24.0 ± 4.1 ml·min^−1^; after 20 min of hemorrhage; *p* < 0.05; Figure 1C). The HSI partially restored this parameter to basal levels in the SHAM A1 group (−10.7 ± 4.2 ml·min^−1^; after 60 min of HSI; Figure 1C) but not the LES A1 group (−21.8 ± 4.5 ml·min^−1^; after 60 min of HSI; Figure 1C).

The lesion of the noradrenergic A1 group did not alter renal blood volume after HSI (SHAM A1: 127.2 ± 13.9 ml and LES A1: 123.3 ± 7.7 ml; between 20 and 60 min of HSI; Figure 1C). Similarly, there were no changes in the redistribution of aortic blood volume (SHAM A1: 1,356.7 ± 205.9 ml and LES A1: 1,130.7 ± 152.6 ml; between 20 and 60 min of HSI; Figure 1C). No changes were observed in the hind limb blood volume of animals from the A1 lesion group when compared with the control (SHAM A1: 1,102.3 ± 178.1 ml and LES A1: 883.6 ± 137.2 ml; between 20 and 60 min of HSI; Figure 1C).

### Effects of A2 Neuronal Lesions on Hypertonic Saline Infusion-Induced Cardiovascular Responses in Rats With Hypotensive Hemorrhage

**Figure 2:**
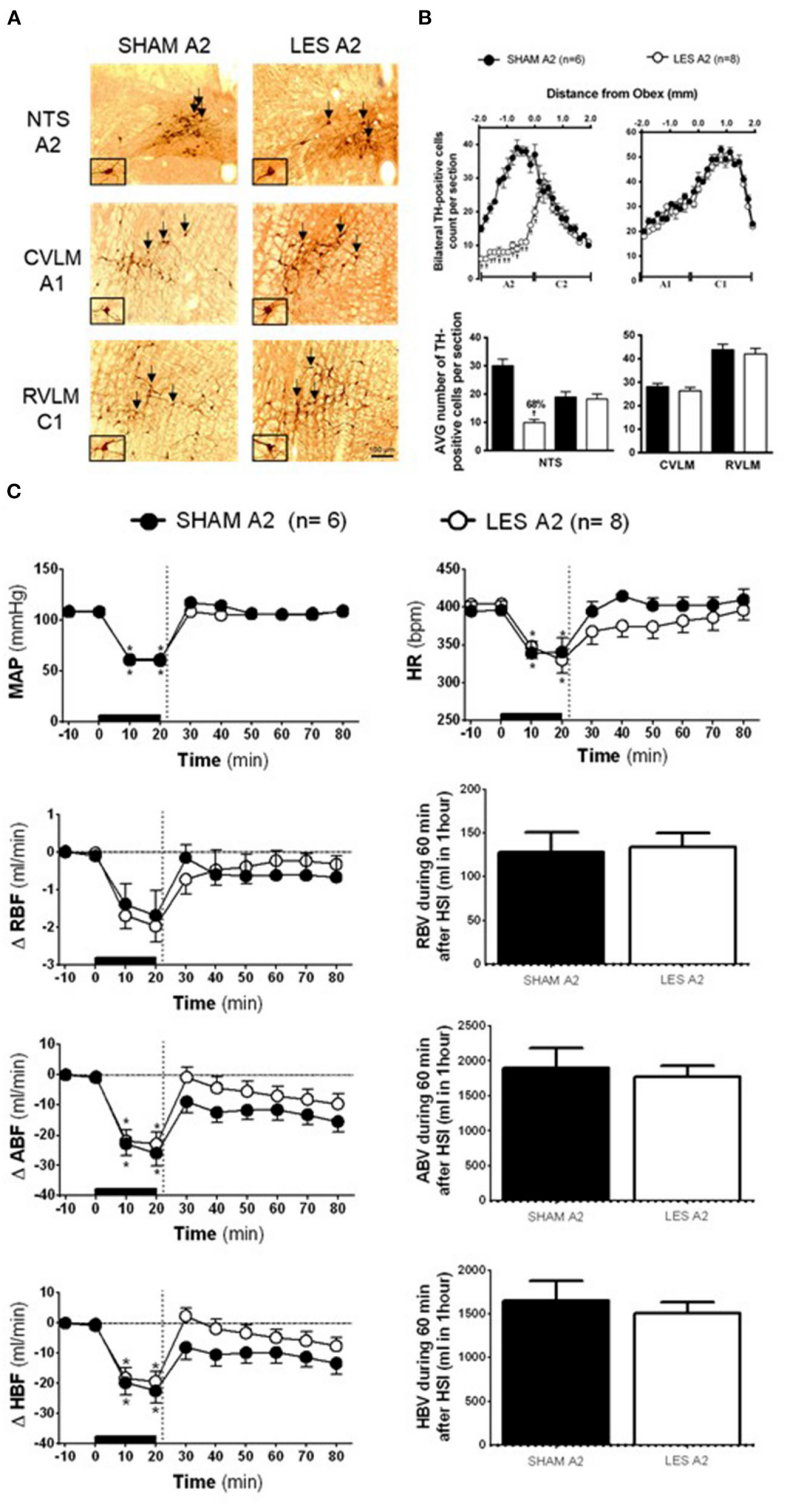
**(A)** A representative photomicrograph of medullary coronal sections (40 μm) with the scale bar of 100 μm. The arrows indicate positive tyrosine hydroxylase neurons in the nucleus of the solitary tract (NTS—cluster A2), caudal ventrolateral medulla (CVLM—cluster A1), and rostrovetrolateral medulla region (RVLM—cluster C1) of the animals receiving nanoinjection of saporin (SHAM A2) or saporin-anti-DβH (LES A2) into NTS. The scale bar is equal to 100 μm. **(B)** Quantification of tyrosine hydroxylase (TH)-positive cells in the medullary regions. The number of positive TH cells in the nucleus of the solitary tract (NTS—clusters A2 and C2), caudal ventrolateral medulla (CVLM—cluster A1), and rostrovetrolateral medulla region (RVLM—cluster C1) of the animals receiving nanoinjection of bilateral saporin (SHAM A2) or saporin-anti-DβH (LES A2) in NTS is expressed as mean ± standard error of the mean (SEM) of *t*. ^†^different from control group with *p* < 0.05. **(C)** The effects of A1 catecholaminergic neuron lesions on changes in mean arterial pressure (MAP), heart rate (HR), variations of renal blood flow (ΔRBF, ml·min^−1^), aortic blood flow (ΔABF, ml·min^−1^), hind limb blood flow (ΔHBF, ml·min^−1^), renal blood volume (RBV), aortic blood volume (ABV), and hind limb blood volume (HBV) during 60 min after hypertonic saline infusion (HSI) in the SHAM A2 (*n* = 6) and LES A2 (*n* = 8) groups submitted to hypotensive hemorrhage. Data expressed as means ± standard error of the mean (SEM) by two-way ANOVA and Bonferroni *post-hoc* test.^†^*p* < 0.05 vs. control group; ^*^p < 0.05 vs. time 0. Black bar and dashed line represent hemorrhagic period and HSI (3 M of NaCl), respectively.

In rats injected with saporin into NTS (SHAM A2; *n* = 6), TH-positive neurons in the region between the obex and 1,800 μm caudal to the obex were averaged 30 cells per section (cluster of A2 neurons). The average number of TH-positive neurons caudal to the obex was reduced to ~10 cells per section in the animals that received bilateral nanoinjections of anti-DβH-saporin into NTS (A2 lesioned group; *n* = 8). This indicates a reduction of ~66% in the cells of the region comprising A2 neurons (Figures 2A,B). The nanoinjections of anti-DβH-saporin in NTS did not reduce the average number of TH-positive neurons per section in the region between the obex and 1,900 μm rostral to the obex region, which includes the group of C2 neurons (Figures 2A,B). No significant changes were observed in the number of TH-positive A1 or C1 neurons in VLM located between 1,800 μm caudal and 1,900 μm rostral to the obex (Figures 2A,B).

Baseline values of cardiovascular parameters MAP, HR, RBF, and ABF were similar in the SHAM A2 and A2 lesioned (LES A2) groups (Table 1). During the hemorrhage, similar blood volume was removed in both groups (SHAM A2: 4.1 ± 0.3 ml vs. LES A2: 4.3 ± 0.5 ml). The hemorrhage-induced hypotension in the SHAM A2 (108.7 ± 5.4 to 60.0 ± 0.6 mmHg) and LES A2 groups (108.3 ± 1.9 to 61.5 ± 1.5 mmHg; 20 min after the beginning of hemorrhage; Figure 2C) was similar. The HSI induced the recovery of MAP close to baseline value in both groups (SHAM A2: 106.5 ± 4.6 mmHg vs. LES A2: 106.1 ± 2.7 mmHg, 30 min after HSI; Figure 2C). This effect was maintained throughout the experiment (SHAM A2: 109.4 ± 3.7 mmHg vs. LES A2: 108.6 ± 5.1 mmHg, 60 min after HSI; Figure 2C). Both groups showed a significant and equipotent bradycardia during hemorrhage (SHAM A2: 340.6 ± 18.4 bpm vs. LES A2: 330.1 ± 17.9 bpm, 20 min after the beginning of hemorrhage; Figure 2C). After 10 min of HSI, HR returned to baseline levels (SHAM A2: 394.4 ± 11.4 bpm vs. LES A2: 367.7 ± 19.9 bpm; Figure 2C).

During hemorrhage, the RBF was reduced in both groups (SHAM A2: −1.7 ± 0.6 ml·min^−1^ vs. LES A2: −2.0 ± 0.4 ml·min^−1^; 20 min after the beginning of hemorrhage; *p* < 0.05; Figure 2C), and the HSI was able to partially restore RBF (SHAM A2: −1.0 ± 0.6 ml·min^−1^ vs. LES A2: −0.4 ± 0.4 ml·min^−1^; 30 min after HSI; Figure 2C). The decrease in the ABF during HH was similar in the SHAM A2 (−25.9 ± 4.1 ml·min^−1^) and LES A2 groups (−22.9 ± 4.0 ml·min^−1^; 20 min after the beginning of hemorrhage; *p* > 0.05; Figure 2C). The HSI restored this parameter to basal level in the SHAM A2 group (−15.5 ± 3.5 ml·min^−1^; 30 min after HSI; Figure 2C) and LES A2 group (−9.7 ± 3.3 ml·min^−1^; 30 min after HSI; Figure 2C). The restorations were not different between groups. The HBF was reduced in both groups during hemorrhage (SHAM A2: −22.5 ± 4.1 ml·min^−1^ vs. LES A2: −19.4 ± 3.4 ml·min^−1^; 20 min after the beginning of hemorrhage; *p* < 0.05; Figure 2C), and the HSI was able to partially restore HBF (SHAM A2: −9.8 ± 3.4 ml·min^−1^ vs. LES A2: −3.3 ± 2.8 ml·min^−1^; 30 min after HSI; Figure 2C). The restorations were not different between groups.

No changes were observed in the renal blood volume of animals from the A2 lesion group when compared with the control (SHAM A2: 128.0 ± 23.1 ml and LES A2: 134.3 ± 16.2 ml; between 20 and 60 min after HSI; Figure 2C). The HSI did not alter the aortic blood volume in the SHAM A2 group (1,903.5 ± 277.5 ml; between 20 and 60 min after HSI; Figure 2C) or LES A2 group (177.4± 158.0 ml; between 20 and 60 min after HSI; Figure 2C). Similarly, there were no changes in the redistribution of hind limb blood volume (SHAM A2: 1,647.5 ± 231.5 ml and LES A2: 1,509.4 ± 126.0 ml; between 20 and 60 min after HSI; Figure 2C).

### Effects of Concomitant Lesion of A1 and A2 Neurons on Hypertonic Saline Infusion-Induced Cardiovascular Responses in Rats With Hypotensive Hemorrhage

**Figure 3:**
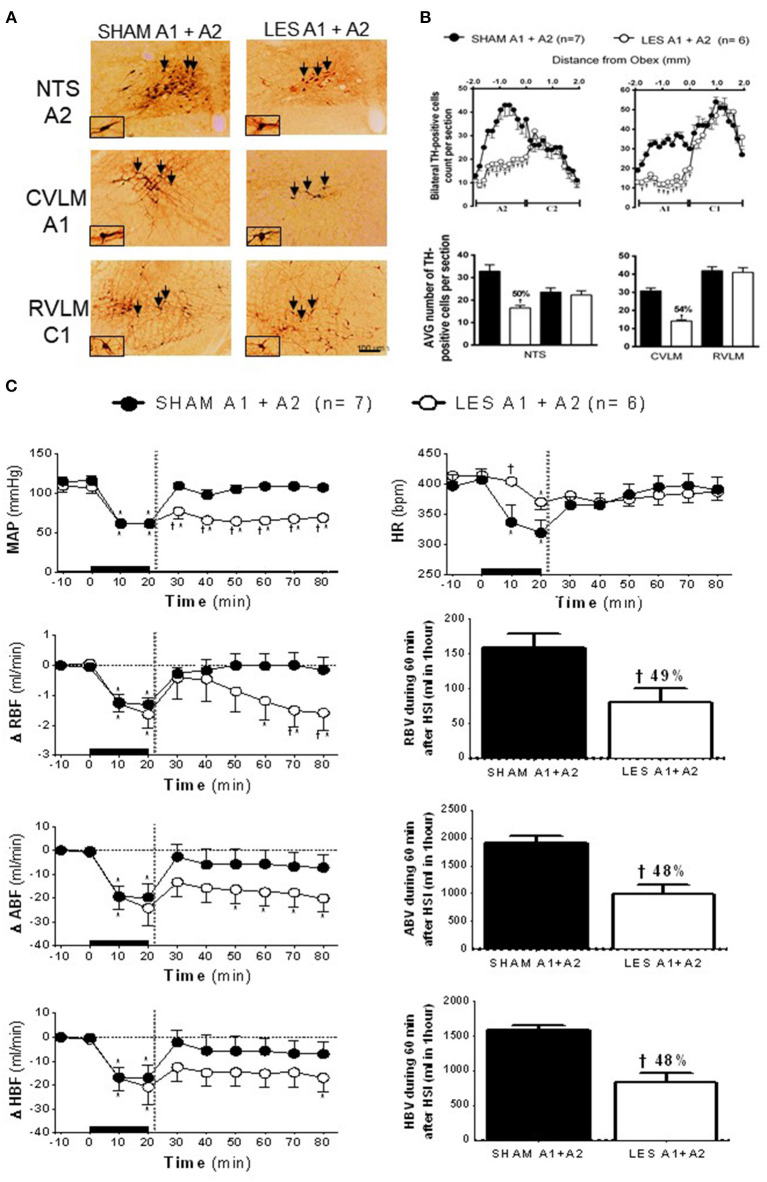
**(A)** A representative photomicrograph of medullary coronal sections (40 μm) with the scale bar of 100 μm. The arrows indicate positive tyrosine hydroxylase neurons in the nucleus of the solitary tract (NTS—cluster A2), caudal ventrolateral medulla (CVLM—cluster A1), and rostrovetrolateral medulla region (RVLM—cluster C1) of the animals bilaterally nanoinjected with saporin (SHAM A1+A2) or saporin-anti-DβH (LES A1+A2) in CVLM and NTS. **(B)** Quantification of tyrosine hydroxylase (TH)-positive cells in the medullary regions. The number of positive TH cells in the nucleus of the solitary tract (NTS—clusters A2 and C2), caudal ventrolateral medulla (CVLM—cluster A1), and rostrovetrolateral medulla region (RVLM—cluster C1) of the animals receiving nanoinjection of bilateral saporin (SHAM A1+A2) or saporin-anti-DβH (LES A1+A2) in CVLM and NTS is expressed as mean ± standard error of the mean (SEM) of *t*. ^†^different from control group with *p* < 0.05. **(C)** The effects of A1 catecholaminergic neuron lesions on changes in mean arterial pressure (MAP), heart rate (HR), variations of renal blood flow (ΔRBF, ml·min^−1^), aortic blood flow (ΔABF, ml·min^−1^), hind limb blood flow (ΔHBF, ml·min^−1^), renal blood volume (RBV), aortic blood volume (ABV), and hind limb blood volume (HBV) during 60 min after hypertonic saline infusion (HSI) in the SHAM A1+A2 (*n* = 7) and LES A1+A2 (*n* = 6) groups submitted to hypotensive hemorrhage. Data are subjected to two-way ANOVA and Bonferroni *post-hoc* test and expressed as means ± standard error of the mean (SEM). *Different from time 0; ^†^different from control group with *p* < 0.05. The black bar and dashed line represent hemorrhagic period and HSI (3 M of NaCl), respectively.

In animals nanoinjected with saporin (non-conjugated) into both the CVLM and NTS (SHAM A1+A2; *n* = 7), the number of TH-positive neurons in the VLM between 200 and 1,900 μm caudal to the obex was about 30 cells per section (cluster of A1 neurons), while the region between the obex and 1,800 μm caudal to the obex averaged 33 cells per section (cluster of A2 neurons). The animals nanoinjected with anti-DβH-saporin into both CVLM and NTS (A1+A2 lesioned group; *n* = 6) showed an average number of TH-positive A1 cells of 14 cells per section (a reduction of 54% when compared with SHAM; Figures 3A,B). The number of TH-positive A2 cells was reduced by nearly 16 cells per section, indicating a decrease of ~50% in comparison with SHAM (Figures 3A,B). No significant changes were observed in the number of TH-positive C2 or C1 cells in the NTS and VLM, respectively (Figures 3A,B). This finding showed the specificity and the efficacy of the lesion approach.

The nanoinjection of saporin did not alter the number of TH-positive neurons of the C2 (NTS) or C1 (RVLM) group. This result demonstrated the confinement of the lesion to the nanoinjection site and confirmed that the lesions of the A1 and/or A2 group were responsible for the cardiovascular responses.

The SAM and A1+A2 lesioned (LES A1+A2) groups showed similar baseline values of MAP, HR, RBF, and ABF (Table 1). During the hypovolemic hemorrhage, the volume of blood withdrawn was similar in both groups (SHAM A1+A2: 3.8 ± 0.4 vs. LES A1+A2: 4.1 ± 0.4 ml). An equipotent hypotension was observed in the SHAM A1+A2 (104.7 ± 5.8 to 61.4 ± 0.4 mmHg) and LES A1+A2 groups (109.1 ± 7.9 to 60.7 ± 0.7 mmHg; *p* < 0.05; Figure 3C) after 20 min of HH. However, HSI did not restore MAP in the group with concomitant lesion at A1 and A2 regions (SHAM A1+A2: 104.9 ± 5.7 mmHg vs. LES A1+A2: 64.2 ± 4.5 mmHg; *p* < 0.05; 30 min after HSI; Figure 3C). Hypotension observed in the LES A1+A2 group during hemorrhage was not reversed (SHAM A1+A2: 107.1 ± 3.3 mmHg vs. LES A1+A2: 68.4 ± 4.2 mmHg; *p* < 0.05; during 60 min after HSI; Figure 3C). In the first 10 min of bleeding, we observed a different response in HR from the two groups (SHAM A1+A2: 336.6 ± 29.8 and LES A1+A2: 404.5 ± 7.4 bpm, 30 min after HSI, Figure 3C). Different chronotropic reactivity was observed in the SHAM A1+A2 group (399.7 ± 17.5 bpm vs. 319.9 ± 20.7; *p* < 0.05; Figure 3C) and mild bradycardia in the LES A1+A2 group (413.6 ± 8.6 bpm vs. 370.2 ± 12.5; *p* < 0.05; Figure 3C) after 20 min of hemorrhage. The HR returned to baseline values after HSI in both groups (SHAM A1+A2: 382.8 ± 17.7 and LES A1+A2: 374.9 ± 10.6 bpm, 30 min after HSI, Figure 3C).

The HH reduced RBF in the SHAM A1+A2 (−1.3 ± 0.2 ml·min^−1^; Figure 3C) and LES A1+A2 groups (−1.6 ± 0.5 ml·min^−1^; *p* < 0.05; Figure 3C) after 20 min of hemorrhage. Similarly, restorations of hemodynamic parameter were observed after HSI in both groups (SHAM A1+A2: 0.0 ± 0.4 ml·min^−1^ vs. LES A1+A2: −0.9 ± 0.7 ml·min^−1^, 30 min after HSI; Figure 3C). However, RBF was not restored in the LES A1+A2 group until the end of the experiments (−1.6 ± 0.6 ml·min^−1^, 60 min after HSI; Figure 3C). The restorations were different between groups (SHAM A1+A2: −0.1 ± 0.3 ml·min^−1^ vs. LES A1+A2: −1.6 ± 0.6 ml·min^−1^, 60 min after HSI; *p* < 0.05; Figure 3C).

The ABF was reduced during HH in the SHAM A1+A2 (−19.6 ± 5.5 ml·min^−1^; 20 min after the beginning of hemorrhage; *p* < 0.05; Figure 3C) and LES A1+A2 groups (−24.1 ± 7.4 ml·min^−1^; 20 min after the beginning of hemorrhage; *p* < 0.05; Figure 3C). HSI promoted a subsequent restoration of this parameter to baseline levels in the SHAM A1+A2 group (−5.8 ± 6.6 ml·min^−1^, 30 min after HSI; Figure 3C). However, in the LES A1+A2 group, HSI restored ABF to baseline in the 1st minutes, but this recovery was not maintained after 30 min HSI (−20.1 ± 5.7 ml/min; 30 min after HSI; *p* < 0.05; Figure 3C). During hemorrhage, the HBF was reduced in both groups (SHAM A1+A2: −17.0 ± 5.4 ml·min^−1^ vs. LES A1+A2: −20.9 ± 7.4 ml·min^−1^; 20 min after the beginning of hemorrhage; *p* < 0.05; Figure 3C), and the HSI restored HBF in the SHAM A1+A2 group (−5.8 ± 6.3 ml·min^−1^; 30 min after HSI; Figure 3C) and LES A1+A2 group (−14.7 ± 5.8 ml·min^−1^; 30 min after HSI; Figure 3C).

In the animals with concomitant lesions of the A1 and A2 clusters, there was a decrease in renal blood volume after HSI when compared with control (SHAM A1+A2: 158.2 ± 20.1 ml vs. LES A1+A2: 80.6 ± 19.7 ml; *p* < 0.05, between 20 and 60 min after HSI; Figure 3C). Reductions in aortic blood volume were also noticeable in LES A1+A2 (SHAM A1+A2: 1,906.8 ± 121.1 ml vs. LES A1+A2: 981.9 ± 184.0 ml; *p* < 0.05, between 20 and 60 min after HSI; Figure 3C). In the LES A1+A2, the hind limb blood volume was reduced (SHAM A1+A2: 1,590.4 ± 61.1 ml vs. LES A1+A2: 820.7 ± 144.0 ml; *p* < 0.05, between 20 and 60 min after HSI; Figure 3C).

## Discussion

The present study validated the hypothesis that hemorrhage decreases MAP, HR, RBF, ABF, and HBF in rats and that HSI promotes recovery. Our findings demonstrated the following: (i) lesion of either A1 or A2 neurons did not alter the hemorrhage- or HSI-induced cardiovascular responses; (ii) simultaneous lesion of A1 and A2 neurons did not alter the hemorrhage-induced cardiovascular responses; and (iii) simultaneous lesion of A1 and A2 neurons attenuated HSI-induced recovery of MAP, RBF, ABF, and HBF as well as the recovery of the volume of aortic, renal, and hind limb blood.

The rapid blood volume reduction during hemorrhage is associated with sudden impairment of venous return, HR, cardiac output, and blood pressure reduction (Ao-ieong et al., 2016). Studies showed that the withdrawal of ~15–25% of the total blood volume was enough to induce and to sustain HH (de Almeida Costa et al., 2009; Amaral et al., 2014). Renal sympathoinhibition observed during HH indicates reduction in vascular resistance and decrease in total peripheral vascular resistance, which probably contributed to the observed hypotension. Our results demonstrate that similar blood volume withdrawal promotes hypotension and bradycardia. In addition, we demonstrate that the HH induced reduction in RBF, ABF, and HBF. Thus, it is suggested that hypovolemia-induced MAP reduction would be partly associated with the reduction in HR and cardiac output.

HSI remains one of the first-line treatments of HH (Velasco et al., 1980; Wade et al., 1991; Kramer, 2003; Krausz, 2006). Studies have shown the reestablishment of the HH-induced cardiovascular impairment by HSI (Velasco et al., 1980; de Almeida Costa et al., 2009; Amaral et al., 2014). After hemorrhage, the administration of HS was able to promote a change in free fluid from the “interstitium” to the capillaries, which results in an increase in blood volume and an improvement in cardiac output, which partly explains the recovery of MAP (Drucker et al., 1981; Whalen et al., 2014). Despite the undoubted efficacy of HSI in recovering hemodynamics, the mechanism underlying hyperosmolarity-induced cardiovascular recovery may not be attributed only to the plasma volume expansion (Walsh and Kramer, 1991), which arises the hypothesis that centrally triggered vasomotor adjustments would be involved. In fact, studies demonstrated that carotid denervation or carotid chemoreceptor inactivation abolished the cardiovascular recovery induced by HSI after hemorrhage (de Almeida Costa et al., 2009).

Indeed, the autonomic adjustments after HSI require an efficient reflex system of sensors (mainly chemoreceptors and baroreceptors), central circuitries, afferences, and efferences (Antunes-Rodrigues et al., 1992; Colombari et al., 2000). According to Pedrino et al. (2011), the specific inactivation of the carotid chemoreceptors abolishes the blood pressure recovery induced by HSI in rats submitted to hemorrhage. These results showed that the HSI could activate neuronal reflex mechanisms to restore the homeostasis. Furthermore, studies show that the injury to A2 and A1 clusters does not alter the baseline values of plasma sodium concentration and blood volume (Pedrino et al., 2012; da Silva et al., 2013). Together, our results suggest that neurons A1 and A2 mediate the neural mechanisms involved in hypertonic recovery in hemorrhagic rats.

The NTS with catecholaminergic neurons (immunopositive neurons to TH: A2 clusters) receives information from afferent carotids and peripheral osmoreceptors and sends them to other regions such as CVLM-A1 (Blessing et al., 1982). These noradrenergic neurons project to MnPO, RVLM, SFO, and PVN that regulate the homeostasis of body fluids (Chan et al., 1995; Kawano and Masuko, 1996; Bienkowski and Rinaman, 2008; Rinaman, 2011). The A1 and A2 neuronal projections communicate changes in plasma osmolarity to hypothalamic regions (Hochstenbach and Ciriello, 1994a,b; Ciriello et al., 1996). The diencephalic regions that are deeply involved in the control of hydro-electrolyte balance receive direct and indirect excitatory projections from the A1 and A2 noradrenergic neurons (Tanaka et al., 1997; Menani et al., 2014a).

The hyperosmolarity-induced activation of sympathetic premotor neurons of PVN underlies autonomic and neuroendocrine responses that reestablish MAP (Llewellyn et al., 2012; Menani et al., 2014a). The A1 neuron lesion and acute hypernatremia reduce natriuretic and hypertensive responses (da Silva et al., 2013). In the present study, only concomitant lesion of A1 and A2 neurons attenuated HSI-induced MAP recovery after hemorrhage. Since the diencephalic region is responsible for setting the neural pathways controlling fluid composition and volume (Tanaka et al., 1997), the attenuation of HSI-induced MAP, RBF, and ABF recovery in hemorrhagic rats with A1 and A2 neuronal lesions confirmed that these neuronal components are essential in the regulation of these adjustments. The integrity of A1 and A2 neuronal pathways connecting peripheral afferents to the hypothalamus is critical to the HSI-induced recovery during hemorrhage. In addition, recent work has shown that the carotid bodies are activated by HSI. The carotid bodies contribute to the increase in sympathetic activity during acute HSI (da Silva et al., 2019).

Since adequate conductance in some arterial territories is essential to maintain tissue perfusion (Kramer, 2003), a decrease in blood perfusion of renal and aortic vascular beds may lead to local ischemia (Kramer, 2003) and irreversible damages. Thus, the functional integrity of the multiple systems regulating vasomotor tone is a limiting step to sustain perfusion in different hemorrhagic conditions (Kramer, 2003). Non-reestablishment of MAP after concomitant lesion of A1 and A2 neurons could be attributed to non-recovery of RBF and ABF after HSI. A lesion of these adrenergic neurons seems to disrupt the connection between peripheral and central circuits. Hence, A1 and A2 are important synaptic relays in the HSI and hemodynamic responses. Therefore, we hypothesized that the attenuation of HSI-induced cardiovascular reestablishment involved direct or indirect disruption of the link among A1, A2, MnPO, SFO, PVN, and SON.

In these experiments, we showed that animals with A1 and A2 lesions did not recover blood flow. Studies with a similar injury time show that the loss of ~60% of A1 and/or A2 neurons is effective in establishing different responses in the control animals (Pedrino et al., 2012; da Silva et al., 2013; Freiria-Oliveira et al., 2015; Naves et al., 2018). Current results consistently demonstrated that lesions in two areas effectively attenuated the HSI-induced recovery. The selective immunotoxin-induced lesion has been reported only in cells that incorporate this complex (Wrenn et al., 1996; King et al., 2015; Malheiros-Lima et al., 2017). In the present study, the lesions were considered specific, as only the animals that received the complex showed a reduction in the number of neurons. The lack of significant difference in the numbers of TH-positive neurons in the region of nanoinjections (RVLM) further supports the selectivity and restriction of lesions in our study.

Although the lesion of the A1 or A2 neuronal groups in isolation did not alter the cardiovascular induced by sodium overload or due to hypovolemia, we cannot exclude parallel central pathways that regulate the same function. The latter assumption is supported by previous studies that demonstrated that the concurrent lesion, the combined lesion of the anteroventral third ventricle and commissural NTS, reduced MAP in spontaneously hypertensive rats while the separate lesion of each nucleus did not induce antihypertensive effects (Moreira et al., 2009). This investigation demonstrated the importance of parallel pathways for cardiovascular control. As the neurons from A1 and A2 clusters share the same embryological origin (Anderson et al., 1997; Hirsch et al., 1998), display similar projections controlling fluid composition and vasomotor such as MnPO and PVN (Tanaka et al., 1997; Menani et al., 2014a), and are similarly responsive to hyperosmotic stimuli (Pedrino et al., 2008, 2012), we suggest that these groups of neurons act as parallel pathways. In order to test this conceptual model, we show that the simultaneous lesion of A1 and A2 cluster neurons has abolished the compensatory mechanism, HSI-induced MAP restoration, and recovery of blood flow after HH. Hence, it seems valid to infer that the concomitant lesion of A1 and A2 neurons is associated with the complete loss of these compensatory responses.

As A1 and A2 cells project to regions (MnPO, PVN, and SFO) involved in hydroelectrolytic adjustments, an injury in these groups could lead to signaling deficit and impairment of the recruitment settings of other pathways before the attenuation of HSI-induced recovery after hemorrhage. It can be reasonably concluded that the loss of HSI-induced recovery probably reflects the time-dependent cellular or circuit adaptations that occur as a result of A1 and A2 cells loss rather than a loss of integrative processing of synaptic signals that cell A1 or A2 would normally have provided.

## Conclusion

Our results show that lesions need to be in both the A2 region of the NTS and A1 region of the CVLM and play important roles in HSI-induced cardiovascular recovery during hypovolemia. Thus, dysfunction of these neuronal clusters could impair the efficacy of HSI-induced recovery after HH.

## Data Availability Statement

The original contributions presented in the study are included in the article/supplementary material, further inquiries can be directed to the corresponding author/s.

## Ethics Statement

The animal study was reviewed and approved by Ethics Committee of the Federal University of Goias (protocol number 034/12).

## Author Contributions

SM: data curation, formal analysis, investigation, methodology, project administration, and writing-original draft. LN: data curation, formal analysis, investigation, methodology, and project administration. TS: methodology. JA: review and editing. KC: writing-review and editing and data analysis. MF-N: funding acquisition, review, and editing. CC: writing-review and editing, funding acquisition, data analysis. AF-O: writing-review and editing, funding acquisition, and data analysis. JF: writing—review and editing. Rodrigo Mello Gomes: writing-review and editing. EC: writing—review and editing. CX: data curation and writing-review and editing. GP: funding acquisition, supervision, conceptualization, and writing.

## Conflict of Interest

The authors declare that the research was conducted in the absence of any commercial or financial relationships that could be construed as a potential conflict of interest.
